# Dynamics of Pulsed-Laser Interaction with Janus Particles

**DOI:** 10.1021/acsphotonics.4c02388

**Published:** 2025-03-12

**Authors:** Alireza Khoshzaban, Alessandro Magazzú, Maria Grazia Donato, Onofrio M. Maragò, Mehmet Burcin Unlu, M. Natali Cizmeciyan, Parviz Elahi

**Affiliations:** † Institute of Biomedical Engineering, Boğaziçi University, 34684 Istanbul, Turkey; ‡ CNR-IPCF, Istituto per i Processi Chimico-Fisici, I-98158 Messina, Italy; § Faculty of Engineering, Özyeğin University, 34794 Istanbul, Turkey; ∥ Faculty of Aviation and Aeronautical Sciences, Özyeğin University, 34794 Istanbul, Turkey; ⊥ Center for Life Sciences and Technologies, Boğaziçi University, 34342 Istanbul, Turkey; # Department of Natural and Mathematical Sciences, Özyeğin University, 34794 Istanbul, Turkey

**Keywords:** optical manipulation, nanosecond-pulsed lasers, thermophoresis, ablation, Brownian motion, particle tracking

## Abstract

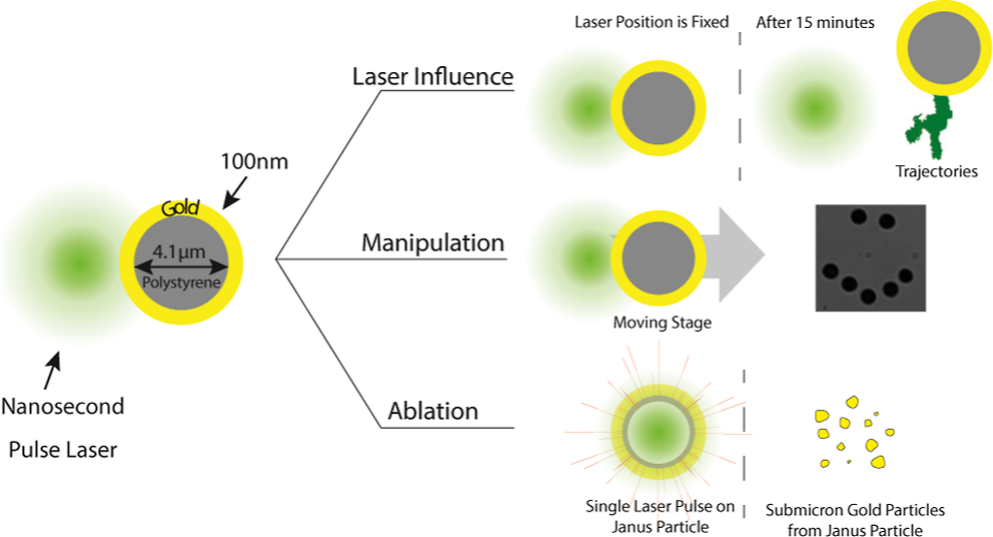

Janus particles, with their flexible chemistry and multifunctionality, have broadened the scope of the optical manipulation field as an emerging class of materials. Laser-based manipulation is particularly promising for half-metal-coated particles, offering a platform to study optical and thermal effects. However, the role of the laser’s operation regime in particle behavior needs to be understood better. Hence, in this work, we studied the interaction of nanosecond-pulsed lasers on 4.1 μm Au-Janus particles with a 100 nm gold cap. We focused on the interaction in three sections: (1) We observed three pulse energy influence regimes: In the low-influence regime (less than ∼10 nJ), the particle maintains its intrinsic Brownian motion. In the medium-influence regime (less than ∼40 nJ), the particle exhibits an extended range of motion. In the high-influence regime (higher than ∼40 nJ), the particle undergoes superdiffusion and establishes a new equilibrium position. (2) During optical manipulation trials, a threshold pulse energy of 4 nJ (average power of 40 μW) was sufficient to move Au-Janus particles against the laser spot. We achieved translation velocities of 0.9–5.1 μm/s at 4–50 nJ. (3) The gold cap is damaged at 20 nJ (fluence of 0.7 J/cm^2^) when the laser is focused on the particle, consistent with theoretical predictions, and the ablation process generates micro- and submicrometer gold particles. These findings reveal the potential of pulsed lasers for precise, power-efficient manipulation of Janus particles, advancing our understanding of laser–particle interactions and opening new pathways for optical manipulation applications.

## Introduction

Janus particles are known for their asymmetry, featuring two distinct surfaces with chemical and physical properties. Active motion on demand makes Janus particles a novel tool for imaging,^1^ drug delivery,^2^ and diagnostics^3−5^ applications. To date, optical, chemical,^6−12^ and physical methods are reported, such as applying external magnetic^7^/electric^13,14^ fields. Optical methods, which involve illuminating particles with a laser, provide the highest flexibility and offer a higher spatial resolution. It is understood that the underlying physical mechanism that initiates the active motion in Janus particles is thermophoresis.^15,16^ In particular, for optical methods, thermophoresis is started by illuminating the Janus particle with light, where only the metallic cap absorbs the light, causing a rapid temperature elevation on the surface, leading to a localized, unsymmetrical, and uneven heating pattern. For nano- and micron-sized Janus particles, temperature differences are easily translated into directed movement.^17−21^ In the literature, many theoretical and experimental studies have been carried out on the optical manipulation of Janus particles using various approaches.^22^ In 2010, Jiang et al.^17^ successfully demonstrated the active motion of Janus particles under laser-induced temperature gradients. Later, the core concept of propulsion was explained by calculating the flow patterns circulating around the Janus particles under the influence of focused laser light.^23^ Further advances in the field have led to the development of techniques such as photon nudging to manipulate the position of microscopic swimmers,^24^ drive nanoparticles,^25^ and microparticle^26^ motors, and with near-infrared light for drug delivery, and controlling the motion of micro- and nanomotors for cargo transport and microfluidics.^27^ However, despite the ability of Janus particles to navigate through random thermal noise and move in a directed manner, a significant challenge still lies in precisely controlling their dynamics. The widespread use of laser-based techniques underlines the need for refined control strategies to harness the potential of Janus particles.

Continuous-wave (CW) lasers are a common tool for manipulating Janus particles, and there are numerous studies in the literature investigating the behavior of Janus particles and controlling them under CW laser illumination.^17,28−30^ In this study, instead of common CW implementation methods, we examined the behavior of Janus particles under a nanosecond-pulsed laser regime. A standard Q-switched laser can generate kilowatts of peak power while maintaining an average power in the milliwatt range.^31^ In pulsed laser interactions, the rapid deposition of energy within short time intervals leads to localized non-steady state heating. Above a certain threshold, rapid temperature elevation leads to the generation of pressure waves,^32^ shock waves,^33^ cavitation,^34^ and ablation,^35^ which might contribute to the propulsion mechanism.

## Results and Discussion

We investigated the interaction between nanosecond-pulsed lasers and Au-Janus particles, concentrating on the influence of the laser on the particles, their optical manipulation properties, and the ablation and damage thresholds of the gold cap. In the first section, we showed that the pulsed laser influences the Brownian motion trajectories of the Au-Janus particles in three regimes. At the so-called high-influence regime, we observed superdiffusivity through mean square displacement (MSD) analysis. In the second section, we explored the novelty of pulsed laser illumination during the optical manipulation of Au-Janus particles. Pulse energies about 4 nJ allowed us to translate the particles with 0.9 μm/s, up to 5.1 μm/s with about 50 nJ of pulse energy. In the final section, we validated our developed theoretical model that beyond 20 nJ, the gold cap melts/damages and generates submicron particles.

The Au-Janus particles utilized in our experiments are 4.1 μm polystyrene microbeads half-coated with a 100 nm thick layer of gold. The schematic of the experimental setup is shown in Supporting Information, Section 1, Figure S1, where the laser beam was focused with a 10× (NA = 0.25) objective lens, and the sample plane was imaged onto the camera with a 100× oil-immersed objective. The laser beam minimum spot size was measured as 2ω_0_ = 2.7 μm using the knife edge method. The movement of the particle is recorded through digital video microscopy at a frame rate of 17 frames per second (FPS). Throughout this article, the experimental setup described above is used for all of the experiments.

### Influence of the Laser on the Au-Janus Particle

**Figure 1:**
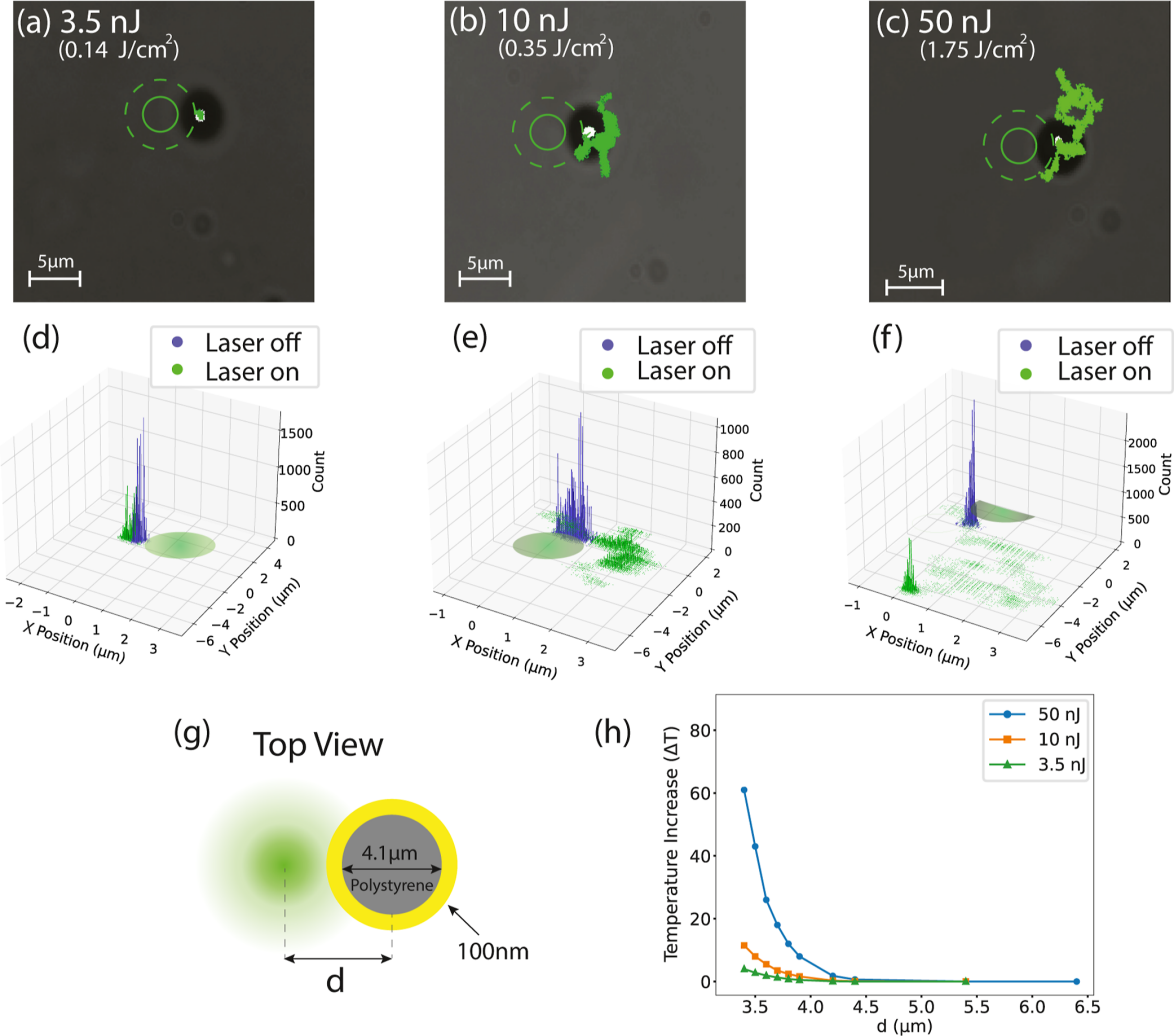
2D trajectory and 3D histograms of a Janus particle under nanosecond-pulsed laser illumination for ω_0_ = 1.35 μm. (a) The low-influence regime at 3.5 nJ, 0.14 J/cm^2^, (b) the medium-influence regime at 10 nJ, 0.35 J/cm^2^, and (c) the high-influence regime at 50 nJ, 1.75 J/cm^2^. In (a–c), the white and green colors show the trajectory of the particle while the laser is off and on, respectively. The green solid and dashed circles show the laser spots at *r* = ω_0_ and *r* = 2ω_0_. (d–f) The corresponding 3D histograms demonstrate how many times the particle was present in a certain coordinate, and the green-colored disk represents the position of the laser relative to the particle. (g) The schematic illustration of the laser spot at a distance *d* to the Janus particle. (h) The simulation result of the temperature increase of the gold Janus particle versus *d* for *E*_P_ = 3.5, 10, and 50 nJ and ω_0_ = 1.35 μm.

We studied the Brownian motion of Au-Janus particles with and without laser illumination. We started by recording the Brownian motion of a particle when the laser was off. After 15 min, we turned on the laser, with a pulse repetition rate of 10 kHz, and illuminated perpendicularly to the left side of the particle, which was recorded for another 15 min. This procedure was repeated at pulse energies of 3.5 nJ and 10–50 nJ. Then, a customized Python code was developed based on OpenCV^36^ to extract the trajectories of the particles after each experiment and plotted in Figure 1a–c. It is important to mention that a 100 nm thick layer of gold makes the particles relatively heavy (the gold cap accounts for 60% of the Janus particle total weight). Due to their weight, particles remain close to the surface of the glass slide, preventing diffusion in the *z* direction.

Trajectories of the particle, shown in Figure 1a–c, allow us to view the collected data comprehensively. 3-D histograms, in Figure 1d–f, of the trajectories show the number of times that particle has been in a specific coordinate, revealing its predominant locations over time. The data suggest three distinct regimes based on the applied pulse energies.

In the first regime, what we call the low-influence regime, when the pulse energy is less than ∼10 nJ (here 3.5 nJ), the particle continues to exhibit its Brownian motion, as indicated by the overlapping trajectories observed under both laser-off and laser-on conditions, as shown in Figure 1a,d. In the second regime, the medium-influence regime (less than ∼40 nJ), at 10 nJ pulse energy, the particle experiences an increased range of motion, seemingly attempting to escape the laser influence, yet it remains in close proximity to the laser zone while still undergoing Brownian motion, as shown in Figure 1b,e. Lastly, in the third regime, the high-influence regime (higher than ∼40 nJ), at 50 nJ pulse energy, the particle experiences superdiffusion which results in pushing the particle far away from the laser beam, establishing a new equilibrium location and continuing its Brownian motion at this new location, as illustrated in Figure 1c,f (see Supporting Information, Section 2, and Video 1).

Dashed circles in Figure 1a–c represent the laser zone, where the laser intensity decreases to approximately 0.03% of its maximum value. To illustrate the functioning of thermophoresis more clearly, we calculated the temperature increase of the gold cap as a function of the distance (*d*) from the center of the laser, as shown in Figure 1g. The results, plotted in Figure 1h, show that at *d*= 3.4 μm, the temperature increases are calculated as 8 °C, 15 °C, and 60 °C for the low-, mid-, and high-influence regimes, respectively. In the high-influence regime, this large temperature gradient causes the particle to settle at a final position approximately *d* = 7 μm away from the laser center. At this new location, sufficiently far from the laser, the effect of thermophoresis diminishes. As a result, the particle undergoes Brownian motion at this new location (see Supporting Information, Section 2 and Video 1).

**Figure 2:**
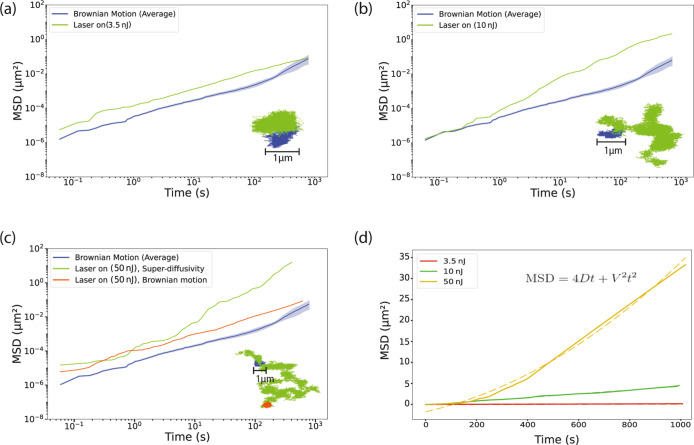
The MSD vs time for three regimes on a log–log scale for pulse energies at (a) 3.5 nJ (low-influence), (b) 10 nJ (medium-influence), and (c) 50 nJ (high-influence). Insets show corresponding particle trajectories. The MSD vs time for three regimes on a (d) linear–linear scale. Dashed lines represent the fitting curves. In the low-influence regime (a), the particle exhibits similar trends in laser on (green line) and off (blue line) states. In the medium-influence regime (b), the MSD slope is steeper when the laser is on (green line) compared to when it is off (blue line), and in the high-influence regime (c), the particle experiences superdiffusion with a parabolic shape MSD (green line) and then relocated in a new location and exhibited Brownian motion (orange line) as if the laser is off (blue line).

We compared the MSD behavior in three different regimes with the case when the laser is off. In this work, we calculated the MSD with respect to the initial position, *r*_0_, as
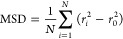
1where *N* is the total number of measurements and *r*_*i*_ is the particle’s position at the *i*th measurement. We used this definition because we wanted to assess how far the particle moves from its starting point under nanosecond-pulsed laser illumination. Figure 2a shows the low-influence regime, where there is no notable difference in MSD between when the laser is on (green line) and off (blue line). As pulse energy increases and approaches the intermediate regime, the slope of the MSD increases (Figure 2b), and the particle exhibits Brownian motion over a broader range, as seen in the inset. In this regime, the particle remains affected by the laser and remains within the laser zone. In the high-influence regime, with the laser on, we initially observed superdiffusive behavior (green line). However, the particle is then pushed out of the laser zone, and once in the new position, it resumes Brownian motion, similar to when the laser is off (Figure 2c).

Figure 2d presents the MSD of the Janus particle at pulse energies of 3.5 nJ, 10 nJ, and 50 nJ. As shown, in the low- and medium-influence regimes, the MSD increases linearly with time, which can be expressed as^37^
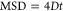
2where *D* represents the particle’s diffusion constant. On the other hand, in the high-influence regime, the MSD follows a quadratic dependence on time and can be described by^17^
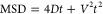
3where *V* is the particle’s self-propelling velocity.

Using eqs 2 and 3 to fit lines on the MSDs shown in Figure 2d highlights the effect of the laser illumination on the particles’ diffusion behavior. In the low-influence regime, at a pulse energy of 3.5 nJ, the MSD versus time shows a diffusion coefficient, *D*, of 0.3 × 10^–4^ μm^2^/s, with no notable difference compared to when the laser is off. As the energy increases, entering the medium-influence regime, the MSD remains linear, while the *D* values rise. At 10 nJ, the calculated *D* is 1.1 × 10^–3^ μm^2^/s. At 50 nJ, the quadratic behavior of the MSD indicates superdiffusion, the particle’s velocity, *V*, is 4.7 × 10^–3^ μm/s, and the *D* is 3.3 × 10^–3^ μm^2^/s.

**Figure 3:**
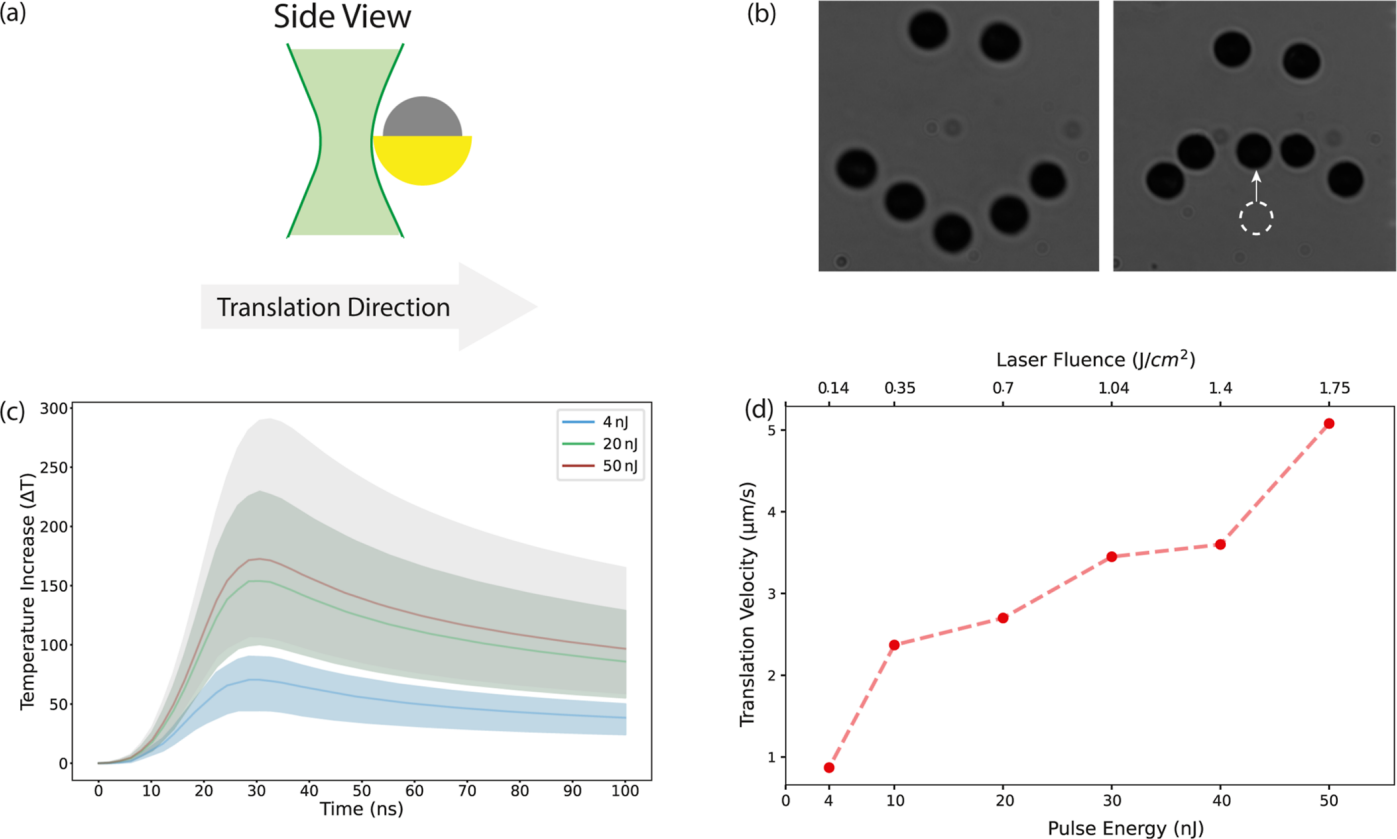
Au-Janus particle manipulation. (a) The schematic of the Janus particle and the laser beam arrangement together with the translation direction. On the side view, solid lines represent the 1/*e*^2^ profile. (b) Janus particles in the desired pattern; the white arrow shows the direction of movement, and the dashed circle represents the initial position. (c) Calculated temperature profiles by considering the minimum and maximum values measured for the separation, *d*, at pulse energies of 4 nJ, 20 nJ, and 50 nJ. (d) The translation velocity of the particles moving together with the laser at given pulse energies and corresponding laser fluences.

In the high-influence regime, the calculated temperature transient of the gold cap reaches 60 °C for tens of nanoseconds. During this transient period (30–100 ns; Figure 3c), the viscosity of the surrounding medium may vary. However, shortly thereafter, the particle stabilizes in a cooler region where the temperature remains constant, owing to the thermal dissipation characteristics of the water medium. Given the experimental time frame—58 ms per frame (with the camera operating at 17 FPS), a total duration of 15 min, and considering that viscosity fluctuations occur on time scales much shorter than those experimentally accessible—we conclude that the average viscosity of the water medium remains effectively constant throughout the measurement period.

### Manipulation of the Au-Janus Particles

In this section, we present our investigation into the optical manipulation of Au-Janus particles using nanosecond-pulsed laser illumination. The experiments were conducted using the same setup detailed in the previous section.

As illustrated in Figure 1g, the laser beam was focused on the particle plane at a distance defined as *d* from the center of the particle. The translational displacement of the Au-Janus particles suspended in the water together with the laser spot (illustrated in Figure 3a) was measured at pulse energies between 3.5 and 50 nJ, with an average power of 35–500 μW, respectively. The threshold pulse energy required to attain translational movement was measured to be approximately 3.5 nJ. We manipulated the position of Janus particles by moving the sample holder stage against the laser spot and arranged them into the desired pattern depicted in Figure 3b. When the laser approached approximately *d* = 3.4 μm to the Janus particle, the laser began to exert influence on it due to the laser absorption (α_gold_ = 5.41 × 10^5^ cm^–1^ at 532 nm). As we push the Janus particle against the laser spot, we observed that depending on the pulse energy of the laser, the separation defined as *d* in Figure 1g has increased. For example, at pulse energies near the threshold, the average separation was *d* = 1.6 ± 0.4 μm. However, at 50 nJ, this distance was raised to *d* = 2.6 ± 0.2 μm. This observation suggests that the particle adjusts its distance from the laser spot based on the applied pulse energy. To see how it affects the maximum temperature elevation, we calculated the temperature increase of the gold cap, taking into account the corresponding separations of *d* measured from recorded movies at pulse energies of 4 nJ, 20 nJ, and 50 nJ (see Supporting Information, Section 4, and Video 2). We used the model we developed based on heat transfer equations (Supporting Information, Section 3) for pulsed laser illumination. The calculated temperature profile over 100 ns is plotted in Figure 3c. Shaded areas correspond to the temperature profiles calculated with minimum/maximum separation values of *d* where the particle is translated together with the laser, and the solid line represents the average temperature elevation profile. From Figure 3c, it is understood that a wide range of temperature elevations are eligible to induce translational movement, starting at a minimum of 50 °C up to 290 °C scaling with incident pulse energy. The pronounced temperature gradient around the particle^26^ drives slip flows due to the tendency of fluids to move from colder to hotter regions,^38^ generating a force that moves the particle in the opposite direction of the temperature gradient.^24^ Later, we estimated the translation velocity of the particles while they stayed coupled with the laser spot from the recorded movies, as shown in Figure 3d. The average elevated temperature of Δ*T* = 170 °C lasted for tens of nanoseconds at 50 nJ of pulse energy and allowed us to translate the particles 5.7 times faster (5.1 μm/s) as opposed to the threshold value of 4 nJ where it was only estimated as 0.9 μm/s (Figure 3d, see Supporting Information, Video 2). Consequently, the interplay between the pulse energy, the induced temperature gradient, and the resulting thermophoretic forces governs both the translation velocity and the particle’s ability to stay coupled with the laser spot.

To show the effective energy deposition ability of pulsed laser illumination, we estimated the required CW laser power to achieve similar temperature elevations acquired in the tens of nanoseconds time range with the pulsed laser. To do that, we have incorporated the expressions derived to estimate the temperature elevation of the gold cap^39^ with a CW laser source (see Supporting Information, Section 4). For example, with 20 nJ of pulse energy, we achieved an average temperature elevation of Δ*T* = 170 °C for 10 ns. According to the expression derived in refs (23 and 39), a CW power of 8.5 mW is necessary to achieve such Δ*T*. Furthermore, we experimentally tried to initiate the movement of Au-Janus particles with a CW laser source using an experimental setup similar to the pulsed laser one. However, even though we exposed the particles to as high as 6 mW of power at 532 nm, we did not observe any discernible movement. Notably, the movement threshold in the pulsed laser was 4 nJ in 10 kHz, corresponding to the average power of 40 μW. The results of the CW experiments demonstrate that a nanosecond-pulsed laser can move the Janus particles with significantly lower average power compared to a CW laser.

We additionally investigated the role of the gold cap in the movement. An uncoated polystyrene (the same polystyrene particles were used to make the Janus particles) was exposed to the same pulse energies. We did not observe any movement in the lateral direction, but a perpendicular movement to the sample plane was observed with pulse energies as high as 700 nJ (7 mW at 10 kHz), indicating the effect of the laser radiation pressure wave pushing the particles in the direction of laser beam propagation.

### Damage Threshold and Ablation of Au-Janus Particles

In this section, we explored the interaction between a single laser pulse and Au-Janus particles with the experimental setup depicted in the Supporting Information, Section 1, Figure S1. The laser pulse duration (FWHM) is 10 ns and focused on the particle. The pulse energies in this experiment ranged from 3.5 to 50 nJ (see Supporting Information, Section 5), similar to what we used in the aforementioned experiments.

**Figure 4:**
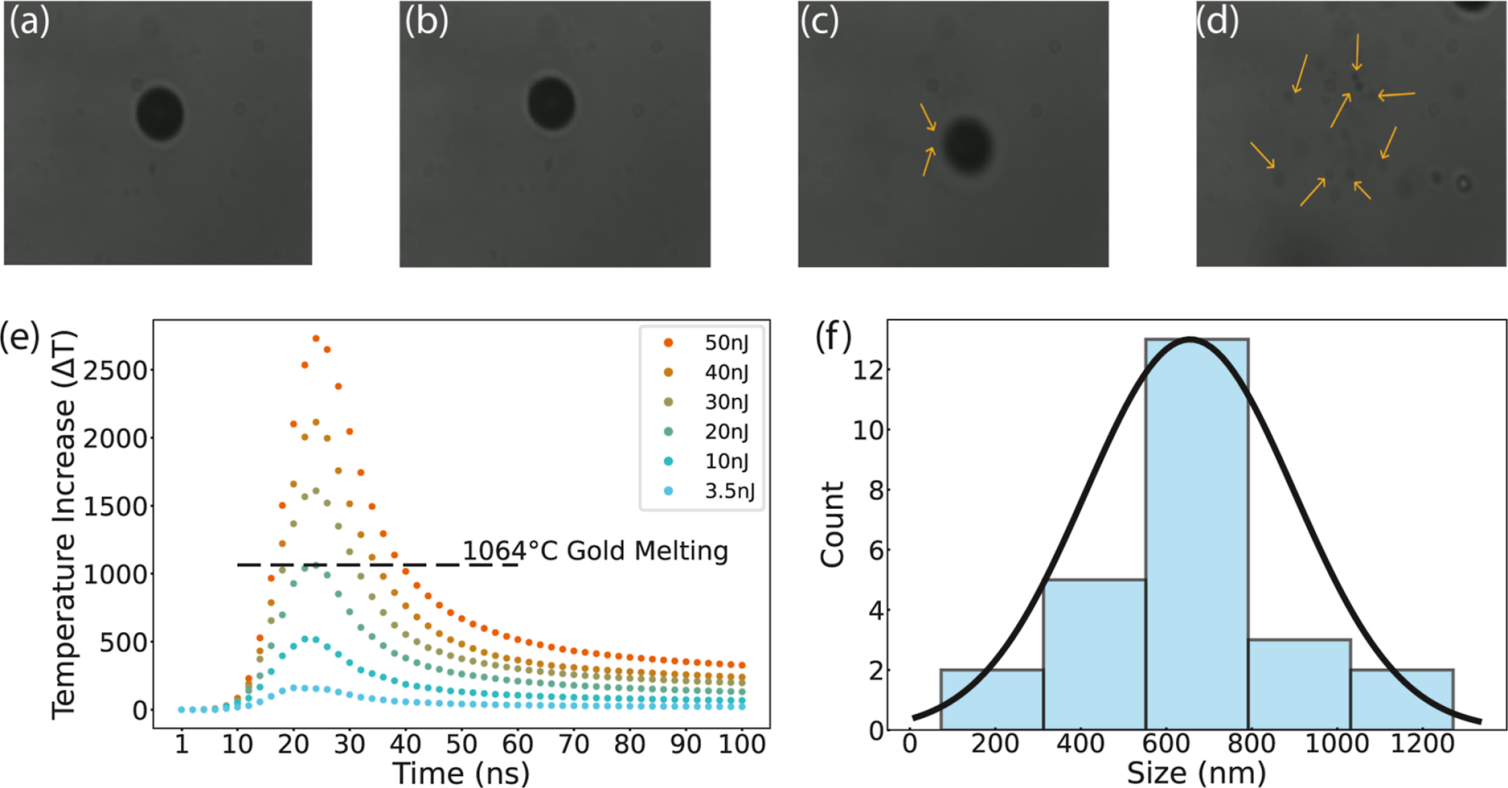
The single laser pulse experiment result. Janus particle (a) before laser illumination and after exposure to pulse energies of (b) 3.5 nJ, (c) 20 nJ, and (d) 50 nJ. (e) Calculated temperature increase of a Janus particle at the center of the laser pulse illumination. (f) Size distribution of gold particles generated by a single 50 nJ, 10 ns pulse with a fluence of 1.75 J/cm^2^.

Figure 4a shows the Au-Janus particle before the laser incidence. Figure 4b–d demonstrates particles exposed to 3.5, 20, and 50 nJ. Figure 4d left the field of view by ejecting the finer particles. We employed a model based on heat transfer equations to calculate the gold cap’s surface temperature after the laser pulse’s arrival. The calculated results in Figure 4e imply that a pulse energy of 20 nJ provides sufficient heating to melt the gold cap, considering the melting point of gold is 1064 °C. Although gold’s absorption is temperature-dependent, its absorption barely changes even when the gold reaches high temperatures under laser illumination at 532 nm.^40^ We carried out experimental tests to validate our theoretical calculation. Focused single laser pulses with 20 nJ of pulse energy (0.7 J/cm^2^) were sent at the Au-Janus particle, and it was observed that debris detached from the particle. We found that the calculated temperatures are consistent with experimental results, Figure 4c.

In 50 nJ of pulse energy (1.75 J/cm^2^), the gold cap’s ablation generates micron and submicron size gold particles,^41,42^ and the particle escapes the field of view (see Supporting Information, Video 3). Figure 4f illustrates the size distribution of these ablated particles in a histogram with a Gaussian function fitted to the data.

## Conclusions

Our study demonstrates the potential of nanosecond-pulsed lasers as an effective tool for manipulating Au-Janus particles with significantly reduced power requirements compared to CW lasers. The study highlights the effectiveness of nanosecond-pulsed lasers for particle manipulation, presenting a novel and efficient alternative to CW lasers. Our study concluded with three sections. We studied the influence of nanosecond pulse lasers on the Brownian motion of the particles. We observed three regimes: in the first regime, the particle’s Brownian motion remains undisturbed; in the second regime, the particle does a broader Brownian motion; and in the third regime, the particle shows a superdiffusive behavior and continues its Brownian motion at a new location. In the second section, we examined the threshold pulse energy needed to initiate the movement of the Au-Janus particle. We found that this threshold pulse energy is 4 nJ. Additionally, we discussed how the minimum distance between the particle and the laser spot changes as the pulse energy varies. Finally, through theoretical calculations and experimental validation, we determined the critical pulse energy required for gold cap ablation and observed the formation of micron- and submicron-sized gold particles. These findings not only validate the precision and versatility of pulsed lasers in particle manipulation but also open new avenues for power-efficient techniques in the design and application of asymmetrical materials.

## Methods

### Janus Particles

The Janus particles were prepared by metal coating one hemisphere with dielectric spherical particles. First, 60 μL of a 5 mg/mL particle suspension was spread on a microscopy slide and allowed to dry. The obtained monolayer was subsequently coated with a 100 nm Au layer using a sputter coating device (Safematic, CCU-010), dispersed in 50 μL of Milli-Q water by sonication, and concentrated to a final volume of 0.5 μL. The dielectric particles used included polystyrene spheres with diameter of 4.1 μm (Microparticles GmbH, batch code: PS/Q-R-KM427).

### Pulsed Laser System Setup

The experimental setup and the schematic are shown in the Supporting Information, Figure S1. The laser (Spectra-Physics, Explorer One) was operating at 532 nm and equipped with an electro-optic Q switch that allowed us to modify the temporal output of the laser on demand. This allowed us to send a single pulse or burst of pulses to the sample to study the interaction between the optical pulses and Janus particles. The laser was focused with a 10× objective lens (Olympus PLN 10× objective NA = 0.25) to a beam waist radius of 1.35 μm, measured by the knife-edge technique, with the objective being underfilled. We sent 4 nJ, 10 nJ, 20 nJ, 30 nJ, 40 nJ, and 50 nJ of pulse energies to the Janus particle. The sample was illuminated by an LED source and imaged with an oil-immersed 100× objective onto the camera (AM-Scope), where the transmitted laser was blocked by a filter (DMLP-550 nm). Finally, we navigated the sample manually with translational stages in *X* and *Y* directions (Thorlabs, XRN25P/M). The samples were prepared by cleaning a glass slide and sticking a double-sided tape mask to the slide. Afterward, 15 μL of the particles was poured onto the glass slide, and finally, a coverslip was put on the glass slide.

### Temperature Calculation

#### Theory

To study the interaction between the Au-Janus particles and the laser, we conducted the Finite Element Method to simulate the heat transfer from the laser to the gold cap of the particles. Therefore, the key equation within our simulations is the heat conduction equation, written in cylindrical coordinates due to the axial symmetry of the system. The formulation of this equation is as follows
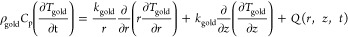
4

where *T*_gold_, ρ_gold_, *C*_p_, *k*_gold_, and *Q*(*r*, *z*, *t*) are defined as the temperature of gold, gold density, specific heat of the gold cap, heat conductivity of gold, and the laser heat generation density, respectively. *Q*(*r*, *z*, *t*) can be written as
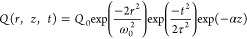
5*r* and *t* represent the spatial and temporal variables, respectively. 2ω_0_ is the beam waist spot size, and τ denotes the standard deviation . α expresses the absorption coefficient of gold, which states how much the beam penetrates the material. See Supporting Information Section 3 for more explanation.

## Supporting Information Available

Temperature calculation, Brownian motion (two laser beam spot sizes), damage threshold, and ablation of Au-Janus particles (PDF)
Brownian motion analysis in pulse energies ranging from 3.5 nJ to 50 nJ (Video 1) (MP4)
Optical manipulation of Au-Janus particles using nanosecond-pulsed laser illumination (Video 2) (MP4)
Ablation of gold layer on Au-Janus particle (Video 3) (MP4)
