# Supplementary material for: Dynamics of Pulsed-Laser Interaction with Janus Particles
Source: ACS Photonics. 2025 Mar 12;12(4):1936–43. doi: 10.1021/acsphotonics.4c02388 (PMC12007091; doi:10.1021/acsphotonics.4c02388)
Supplement: Supplementary file 1 — ph4c02388_si_001.pdf [file ph4c02388_si_001.pdf]

Supporting Information
Dynamics of Pulsed-Laser Interaction with Janus Particle

Alireza Khoshzaban,[†] Alessandro Magazzù,[‡] Maria Grazia Donato,[‡] Onofrio M Maragò,[‡] Mehmet Burcin Unlu,[¶,§] M. Natali Cizmeciyan,[*,‖,¶] and Parviz Elahi[*,⊥]

†*Institute of Biomedical Engineering, Boğaziçi University, 34684 Istanbul, Türkiye*

‡*CNR-IPCF,Istituto per i Processi Chimico-Fisici,I-98158 Messina,Italy*

¶*Faculty of Engineering, Özyeğin University, 34794 Istanbul, Türkiye*

§*Faculty of Aviation and Aeronautical Sciences Özyeğin University, 34794 Istanbul, Türkiye*

‖*Center for Life Sciences and Technologies, Boğaziçi University, 34342 Istanbul, Türkiye*

⊥*Department of Natural and Mathematical Sciences, Özyeğin University, 34794 Istanbul, Türkiye*

E-mail: natali.sozudogru@bogazici.edu.tr; parviz.elahi@ozyegin.edu.tr

1-Experimental Setup

Figure S1 presents a schematic of the experimental setup. A 10X objective and a convex lens (f=5 mm) were incorporated into the optical path to achieve two laser beam spot sizes, as illustrated in Figure S1. The laser beam spot sizes were measured using the knife-edge technique, yielding ω_0=1.35 µm for the 10X objective and ω_0=2.8 µm for the f=5 mm convex lens.

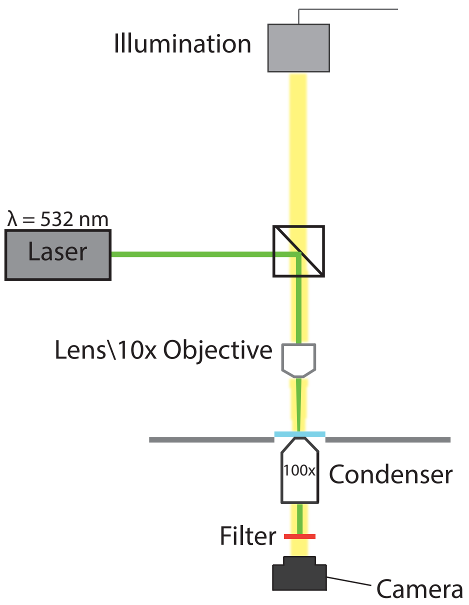

Figure S1: Schematic of the experimental setup. The laser beam was focused with a 10X objective/convex lens (f=5 mm) on the sample plane, and a 100X objective collect the images from the sample plane. A red filter is also used in some experiments to block the laser from entering the camera.

2 - Influence of the Laser on the Au-Janus particle

This section is dedicated to investigating how the laser beam influences the Brownian motion of the particle at a distance defined by d. For this experiment, the particles were recorded for 15 minutes, and then the laser having a spot size of ω_0=1.35 µm turned on the left side of the particle with the initial distance of d_0=3.4 µm and then recorded for 15 minutes again. After we completed this, we repeated the same procedure for approximately two times larger laser

beam spot size ω_0=2.80 µm with the initial distance of $d_0 = 4.8$ µm. Since the laser beam has a Gaussian beam profile, depending on d, the gold part of the Janus particle experiences an effective energy transfer. The effective energy transfer (E_t) to the Janus particle is defined as

$$E_t = \int_d^\infty \frac{2E_0}{\pi\omega_0^2} \exp\left[-\frac{2r^2}{\omega_0^2}\right] 2\pi r\, dr, \tag{1}$$

where d is the distance from the center of the particle to the center of the beam, and E_0 is the incident pulse energy. The transferred energy as a function of d and ω_0 becomes:

$$E_t = E_0 \exp\left[-\frac{2d^2}{\omega_0^2}\right]. \tag{2}$$

To demonstrate how strong this dependence is, we calculated the energy transfer (E_t) and resulting temperature increase (ΔT) with respect to the beam waist spot size (ω_0) and plotted in Figure S2a for E_0=50 nJ. As ω_0 increases, due to the fixed d_0=3.4 µm, the effective energy transfer increases because of a larger overlap with the laser spot, which in turn raises the temperature of the gold cap.

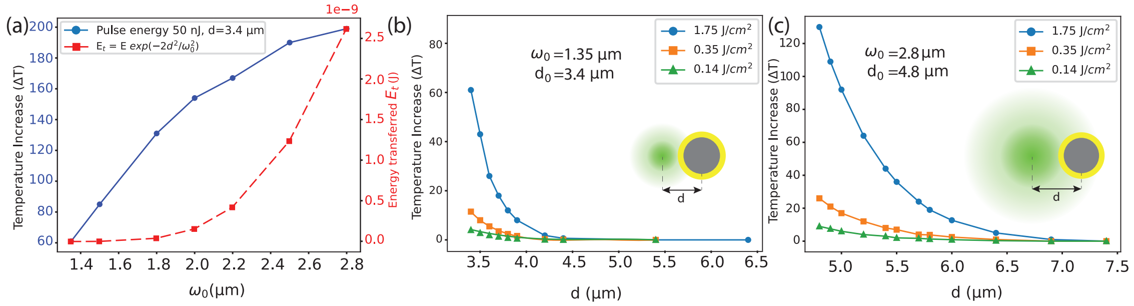

Figure S2: (a) The calculated temperature increase (ΔT) and transferred energy (E_t) versus laser beam spot size (ω_0) at E_0=50 nJ and d_0=3.4 µm. The temperature increase (ΔT) versus (b) d at given laser fluences (J/cm^2) for ω_0=1.35 µm and (c) ω_0=2.8 µm.

Building on this, we further investigated the effect of the laser beam waist spot size on particle trajectories and range of motion. The calculated temperature increase (ΔT) with respect to d for two laser beam waist spot sizes: ω_0=1.35 µm (Fig. S2b) and ω_0=2.8 µm (Fig. S2c), indicates the region where thermophoresis has an influence on the Janus particle. As observed in these figures, a larger spot size with the same laser fluence (J/cm^2) effectively increases the temperature of the gold cap over a broader area.

To further analyze the impact of spot size on particle motion, the following sections will present recorded particle tracking data separately: results for ω_0=1.35 µm in Section 2.1 and for ω_0=2.8 µm in Section 2.2.

2.1- Influence of Laser Beam Spotsize of $\omega_0 = 1.35 \mu m$

The plot of the trajectories Figure S3 and 3D histogram plots Figure S4 reveals three different behaviors for the Janus particles. At 3.5 nJ, the particle does not show any significant change in its motion. At 10-30 nJ, the Janus particle motion shows that the range of the Brownian motion increases, and at 40-50 nJ, the Janus particle escapes from the laser zone and settles in a new location, and continues Brownian motion.

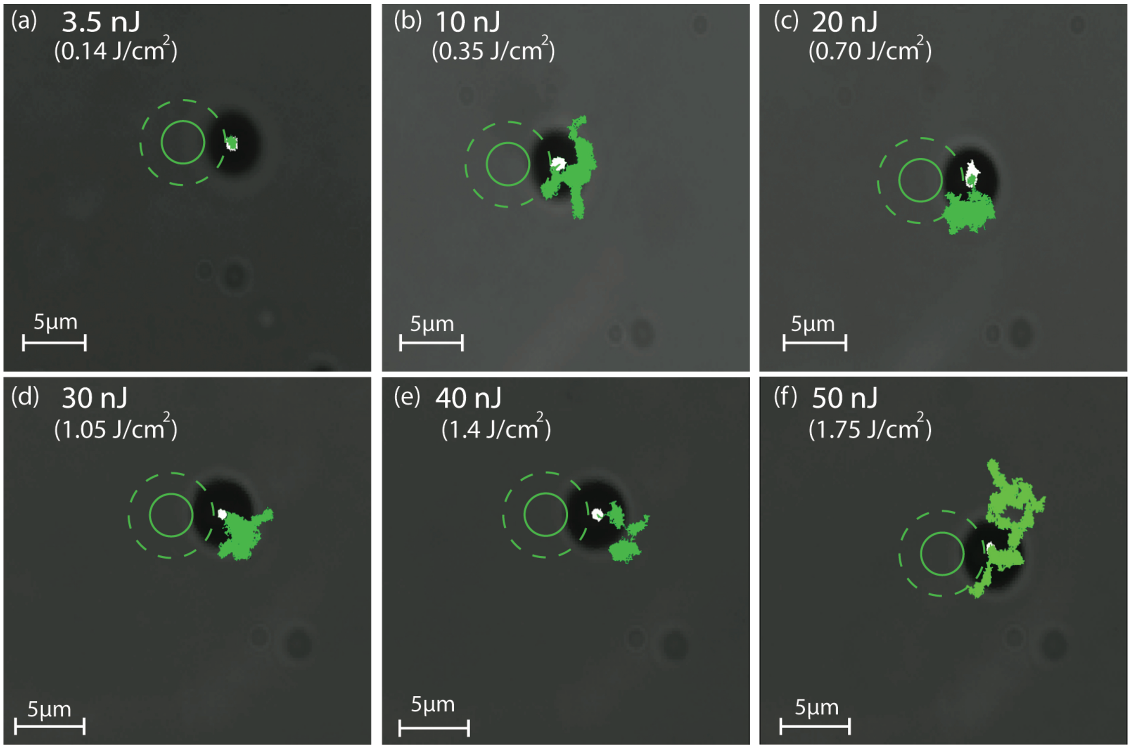

Figure S3: Janus particle Brownian motion analysis in pulse energies ranging from 3.5 nJ to 50 nJ. White lines show the trajectory of the particle while doing Brownian motion. The Green lines show the trajectory of the particle when the laser is present on the left side of the particle (The green circle shows the location of the laser spot)

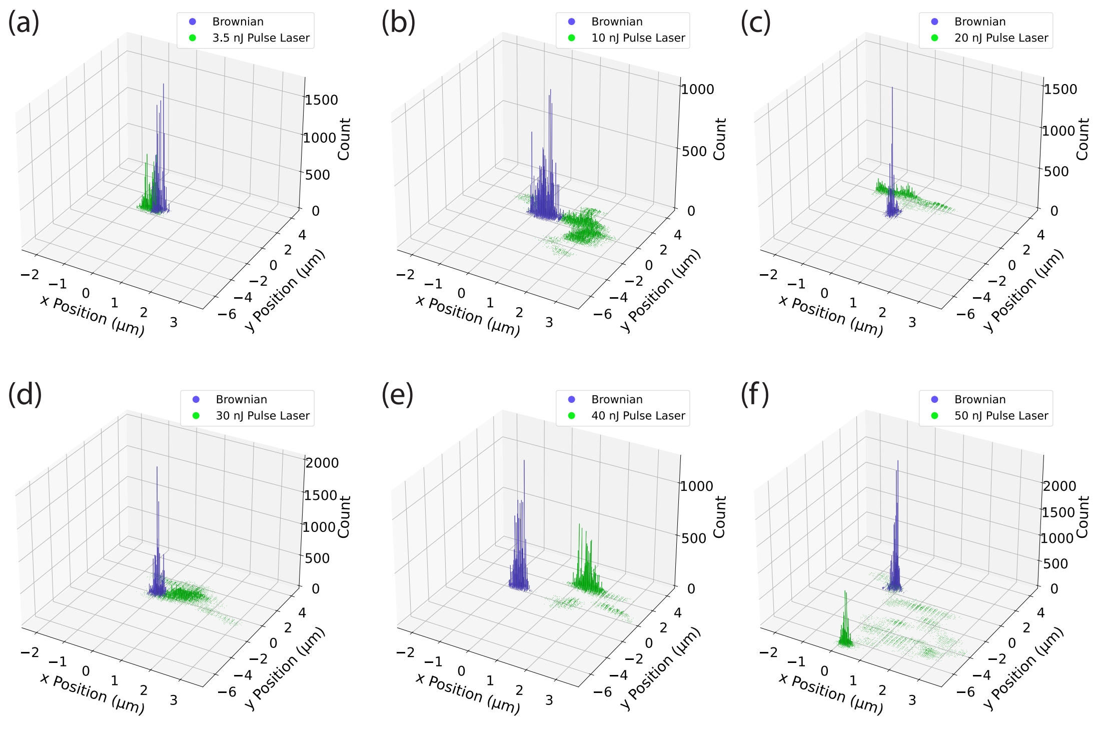

Figure S4: 3D histograms of the trajectories in Figure S3.

The mean square displacement (MSD) of the particles above is plotted in Figure S5. The average of all the Brownian motions when the laser is off is shown in black. Figure S5a shows a log-log scale to highlight the differences in the behaviors in Brownian motion.

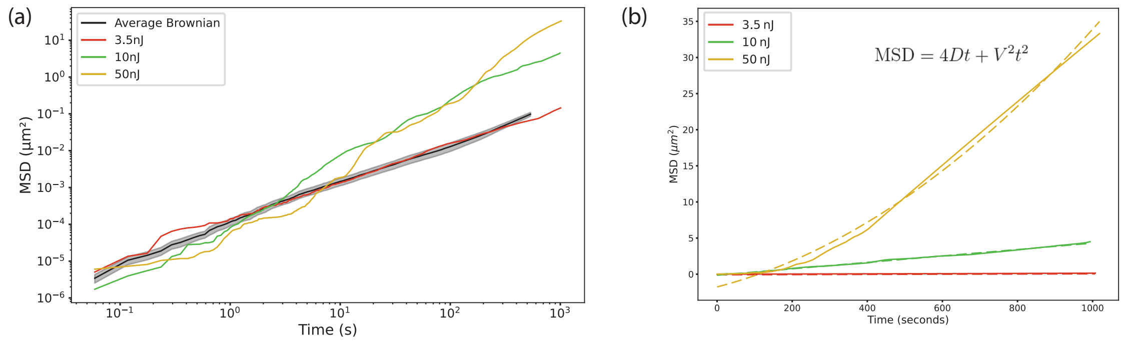

Figure S5: MSD of the Janus particles. 3.5 nJ and the average of Brownian motion have almost the same slope, showing that the laser did not affect the particle Brownian motion. For 10 nJ, the slope is steeper than 3.5 nJ, and for 50 nJ, the line goes parabolic, indicating the super-diffusivity state in (a) log-log scale and (b) linear-linear representation.

2.2 - Influence of Laser Beam Spotsize of $\omega_0 = 2.8\mu m$

We repeated the experiments from Section 2.1 using a laser spot size approximately twice as large (ω_0=2.8 µm) while keeping the laser fluence constant. The 3D histogram data for both spot sizes are plotted in Figure S6. In both cases, the particles exhibit similar histogram counts corresponding to low-, medium-, and high-influence regimes. Additionally, the larger spot size allowed the particles to travel farther from their initial positions. The MSD curves obtained from particle tracking data presented in Figure S7 further confirm that a larger spot size extends the range of motion, highlighting the role of thermophoresis in the movement of the Janus particle.

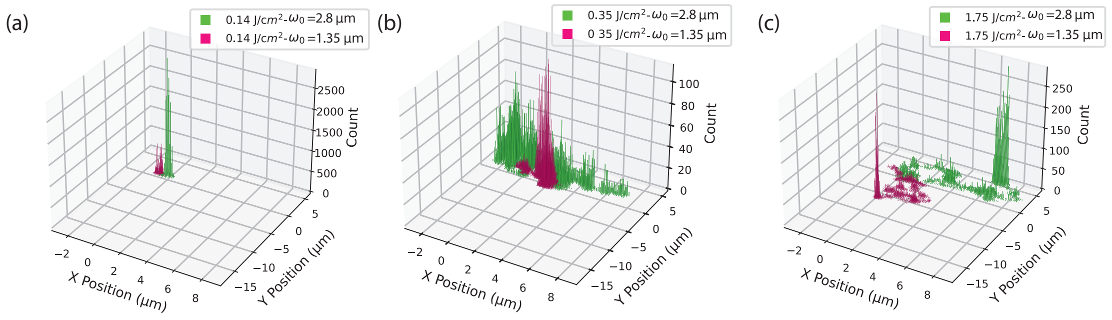

Figure S6: 3D histogram in three regimes (a) low- (b) medium- and (c) high-influence regime for (green) ω_0=2.8 µm and (red) ω_0=1.35 µm.

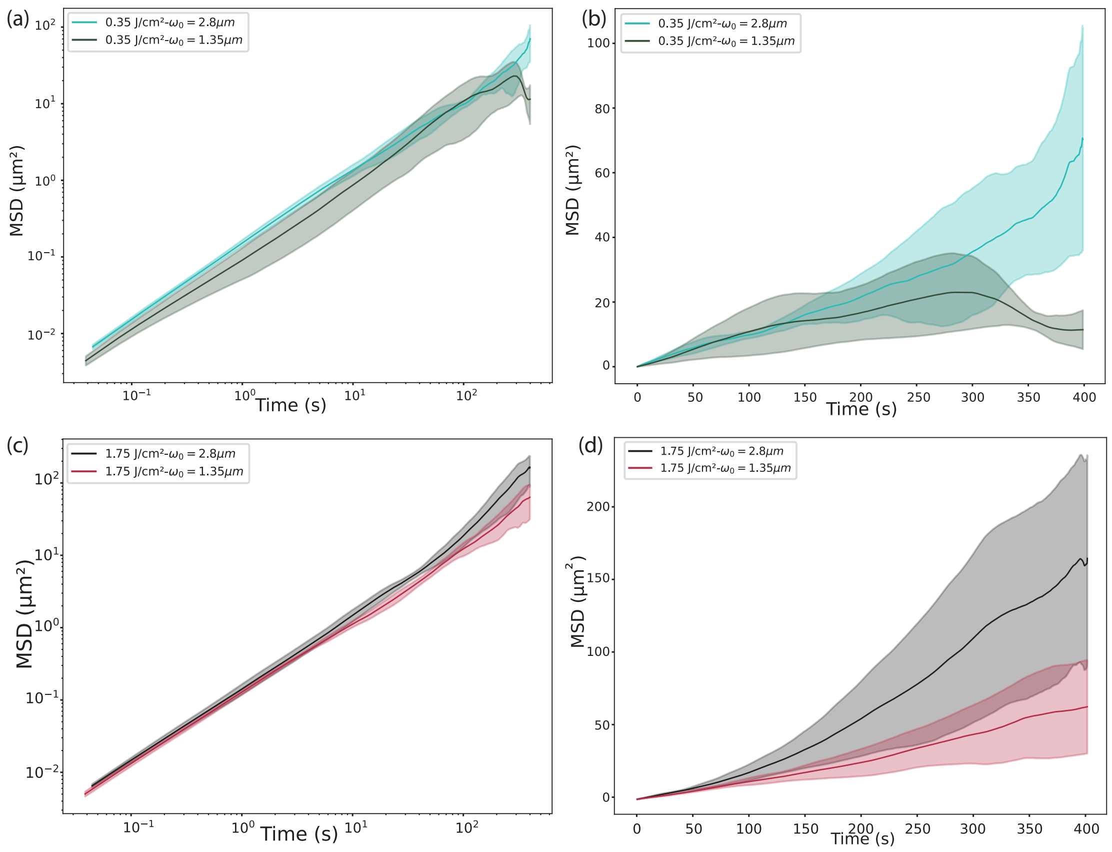

Figure S7: MSD vs time plots of the Janus particles for spot size comparison in log-log scale for (a) laser fluence of 0.35 J/cm^2 and (c) 1.75 J/cm^2 and in linear scale for (b) laser fluence of 0.35 J/cm^2 and (d) 1.75 J/cm^2.

3-Temperature Calculation

The heat that is generated by the laser can be written as:

$$Q(r, z, t) = Q_0 \exp\left(\frac{-2r^2}{\omega_0^2}\right) \exp\left(\frac{-t^2}{2\tau^2}\right) \exp(-\alpha z) \tag{3}$$

By integrating equation 3, we have

$$\int\int Q\,dV\,dt = E_{\text{Pulse}} \tag{4}$$

E_{Pulse} designates the pulse energy of the laser. By substituting equation 3 into equation 4, the integration yields

$$Q_0 \int_0^{2\pi} d\phi \int_0^{\infty} r \exp\left(-\frac{2r^2}{\omega_0^2}\right) dr \int_0^{\infty} \exp\left(-\frac{t^2}{2\tau^2}\right) dt \int_0^{l} \exp(-\alpha z)\,dz = E_{\text{Pulse}} \tag{5}$$

Since $\alpha l << 0$

$$Q_0 = \frac{2E_{\text{Pulse}}\,\alpha}{\pi\omega_0^2\tau\sqrt{\frac{\pi}{2}}} \tag{6}$$

The final form of the $Q(r, z, t)$ is

$$Q(r, z, t) = \frac{2E_{\text{Pulse}}\,\alpha}{\pi\omega_0^2\tau\sqrt{\frac{\pi}{2}}} \exp\left(\frac{-2r^2}{\omega_0^2}\right) \exp\left(\frac{-t^2}{2\tau^2}\right) \exp(-\alpha z)(1 - R_{\text{ref}}) \tag{7}$$

R_{ref} is the reflection coefficient.

Upon formulating the heat conduction equation and the laser heat generation density, it's essential to define the boundary conditions for the simulation. These conditions help govern the temperature changes at the interfaces of different materials within our system.

The boundary conditions used in this simulation are as follows:

On the gold cap and water boundary at $r = R + l$ and $-\pi < \theta < 0$:

$$k_{\text{gold}} \overrightarrow{\nabla} T_{\text{gold}} \; = k_{\text{water}} \overrightarrow{\nabla} T_{\text{water}} + h(T_{\text{water}} - T_{\text{initial}}) + \epsilon_{\text{gold}} \sigma (T_{\text{water}}^4 - T_{\text{initial}}^4) \tag{8}$$

Where ϵ_{gold} is the emissivity of the gold cap, σ is the Stefan-Boltzmann constant, and is the heat transfer coefficient, and R is the radius of the particle and l is the thickness of the gold cap.

On the gold cap and core at $r = R$ and $-\pi < \theta < 0$:

$$k_{\text{gold}} \overrightarrow{\nabla} T_{\text{gold}} = k_{\text{core}} \overrightarrow{\nabla} T_{\text{core}} \tag{9}$$

On the core and water at $r = R$ and $0 < \theta < \pi$:

$$k_{\text{core}} \overrightarrow{\nabla} T_{\text{core}} = k_{\text{water}} \overrightarrow{\nabla} T_{\text{water}} + h(T_{\text{water}} - T_{\text{initial}}) \tag{10}$$

Finally, the temperature of water at the borders of the water container is set to room temperature (298K).

Table 1 below shows the values used in the calculation.

Table 1: Properties and values required for numerical analysis

Property	Symbol	Value	Reference
Reflection coefficient	R_{ref}	0.76	[42]
Absorption coefficient	α	5.41×10^5 ($\frac{1}{\text{cm}}$)	[1]
Pulse duration	τ	10 ns	
Beam waist	ω_0	1.3 μm	
Pulse energy	E_{pulse}	3.5 nJ, 10 nJ, 20 nJ, 30 nJ, 40 nJ, 50 nJ	
Heat conductivity of gold	κ_{gold}	314 $\frac{W}{m\,K}$	[2]
Heat conductivity of water	κ_{water}	0.6 $\frac{W}{m\,K}$	[2]
Emissivity of gold	ϵ_{gold}	0.3	
Stefan-Boltzmann constant	σ	5.67×10^{-8} ($\frac{W}{m^2\,K^4}$)	[3]

Figure S8 depicts two laser incident orientations on the gold cap. In the Brownian motion analysis and manipulation of the Janus particles, the laser illuminated the side of the Janus particles, and in the damage threshold studies, the laser illuminated the center of the gold cap.

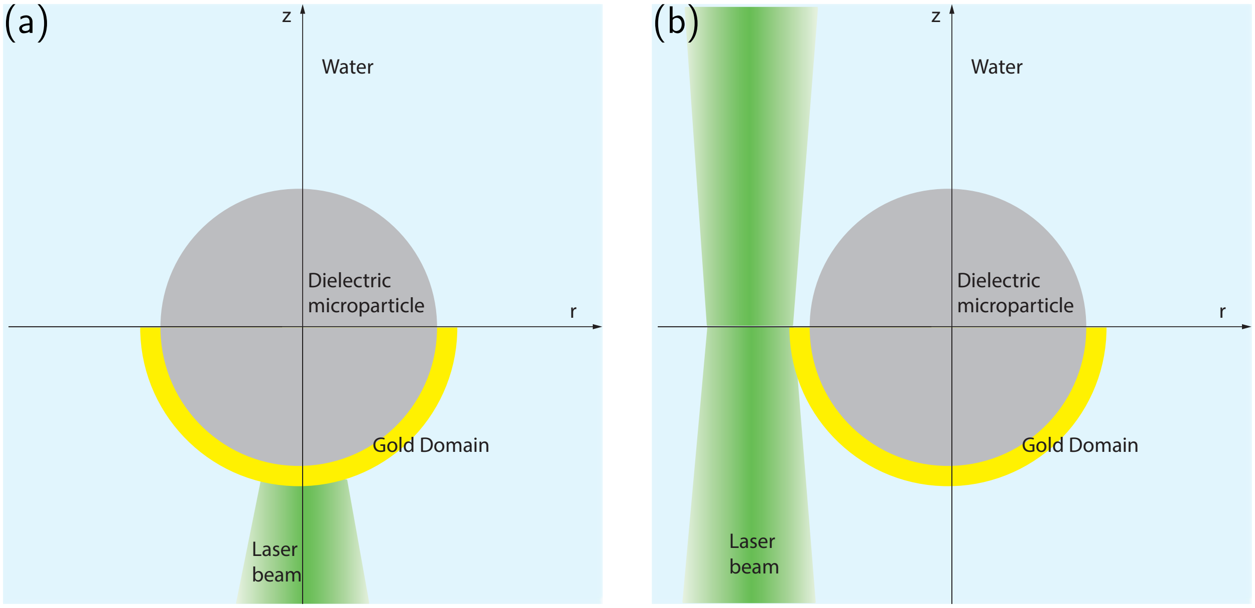

Figure S8: The orientation of the laser beam and gold cap of the Janus particle in the simulation. (a) the laser illuminates the center of the gold cap, (b) the laser illuminates the side of the gold cap.

4-Manipulation of Au-Janus particles

Figure S9(a) shows the effect of distance on the temperature elevation with 4 nJ of pulse energy. Figure S9(b) illustrates the temperature variations with different pulse energies, maintaining a constant distance of 3.4 µm between the particle and the laser spot in the simulation. Figure S9(c) shows the distance of the particle and the center of the laser spot in a trial. The distance varies according to the pulse energy, as shown in Table 2.

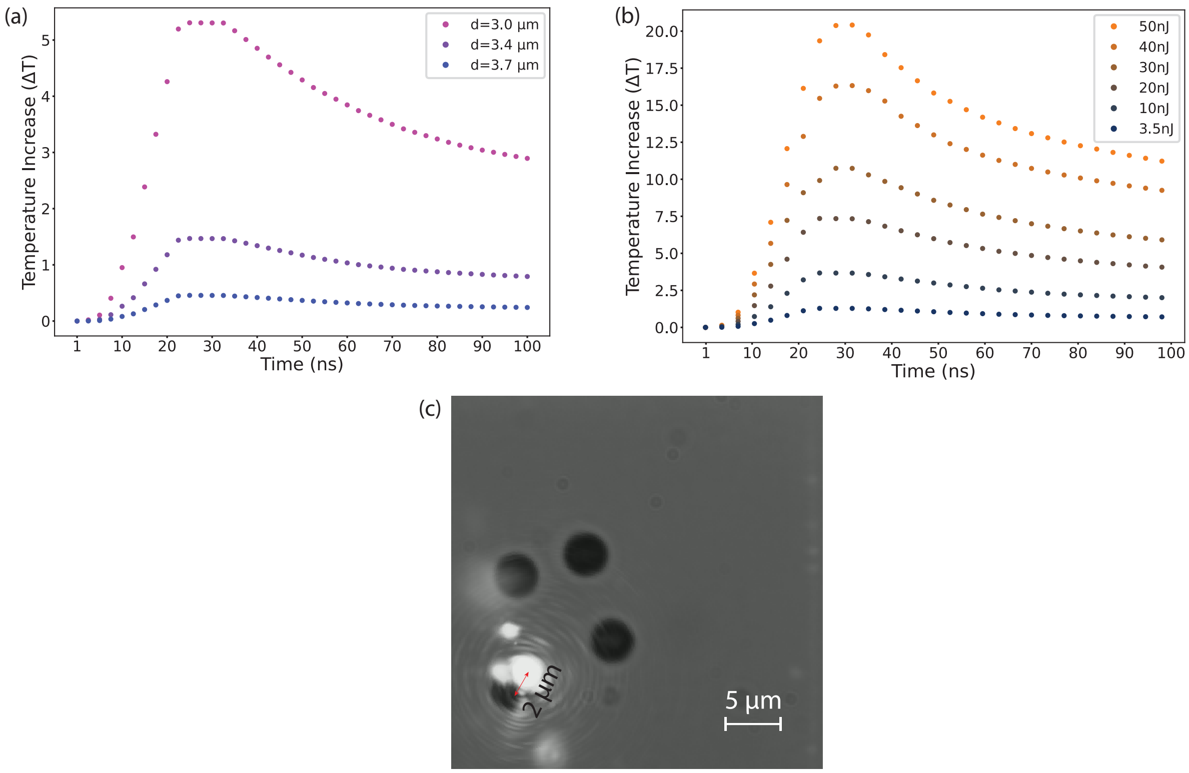

Figure S9: (a) The temperature of the Janus particles varies based on their distance to the laser beam. (b) The temperature increase of the Janus particle when the distance between the laser and particle is 3.4 µm. (c) The distance from the particle to the center of the laser spot was recorded during a trial.

Table 2: distance between the particle and the center of laser spot

Pulse Energy	Average Distance	minimum/maximum
4 nJ	1.6μm	0.4
20 nJ	2.2μm	0.2
50 nJ	2.6μm	0.2

To calculate the temperature of the Au-Janus particle under CW illumination, We used the equation below.[4]

$$P = 2(2+\pi)\kappa_{\text{water}} R \Delta T \tag{11}$$

where κ_{water} (0.6 W/m.K) is the heat conductivity of water, R (2.05 μm) is the radius of the particle, P is the power absorbed by the metal cap. When we place all the known values to equation 11, the required power (P) to elevate the temperature between 100-200°C is estimated to be 5-10 mW.

5-Damage Threshold and Ablation of Au-Janus Particle

This experiment also uses 3.5 nJ, 10 nJ, 20 nJ, 30 nJ, 40 nJ, and 50 nJ of pulse energies. In figure S10, the laser effect on the particles. The "Laser Off" column shows the Janus particle right before the laser incident. The middle column, "Right After Laser Pulse," shows the Janus particle exactly after the laser incident, and in the third column, "Long After Laser Pulse," the particle's image is taken one second after the laser incident.

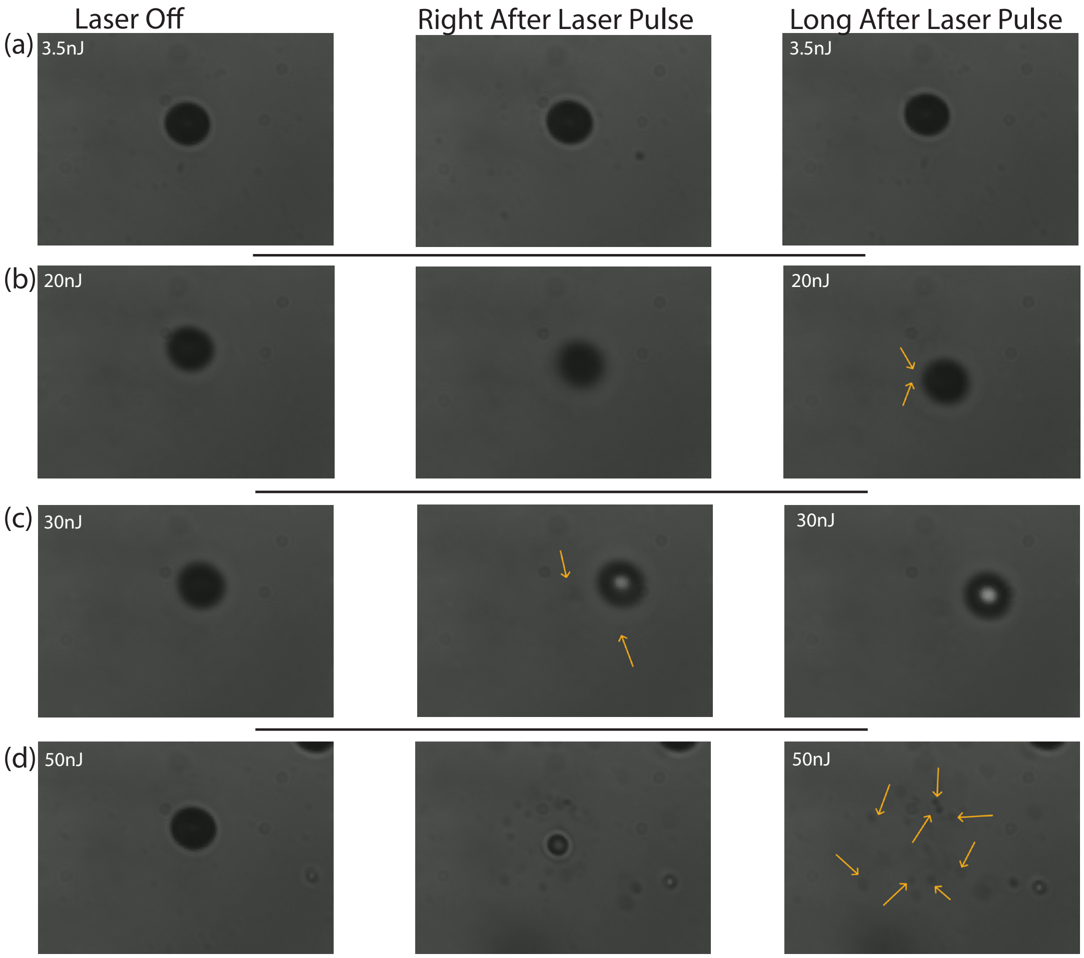

Figure S10: The single laser pulse experiment. Particles are shown when the laser is off, right after, and long after turning on the laser.

References

(1) Yakubovsky, D. I.; Arsenin, A. V.; Stebunov, Y. V.; Fedyanin, D. Y.; Volkov, V. S. Optical constants and structural properties of thin gold films. *Opt. Express* **2017**, *25*, 25574–25587.

(2) of Standards, N. I.; Technology *Security Requirements for Cryptographic Modules*; 2001.

(3) Kolev, N. *Multiphase Flow Dynamics 5: Nuclear Thermal Hydraulics*; Springer International Publishing, 2016.

(4) Bickel, T.; Majee, A.; Würger, A. Flow pattern in the vicinity of self-propelling hot Janus particles. *Physical Review E - Statistical, Nonlinear, and Soft Matter Physics* **2013**, *88*.
